# Exploring the role of generative AI in international students’ sociocultural adaptation: a cognitive-affective model

**DOI:** 10.3389/frai.2025.1615113

**Published:** 2025-06-30

**Authors:** Huajun Ma, Qingnan You, Zhiyuan Jin, Xinglin Liu, Zimeng Chen

**Affiliations:** ^1^College of Wealth Management, Ningbo University of Finance and Economics, Ningbo, China; ^2^School of Business Administration, Zhongnan University of Economics and Law, Wuhan, China; ^3^School of Economics, Shenzhen Polytechnic University, Shenzhen, China; ^4^School of Management, Lanzhou University, Lanzhou, China

**Keywords:** generative AI, sociocultural adaptation, positive reappraisal, perceived, AI anthropomorphism

## Abstract

Against the backdrop of increasing global educational exchanges, the sociocultural adaptation of international students has attracted significant attention. The rise of Generative Artificial Intelligence has brought new perspectives to research in this field, yet existing studies have insufficiently explored the mechanisms through which GenAI influences the sociocultural adaptation of international students. Drawing on the cognitive-affective personality system theory and conservation of resources theory, this study employed a three-stage time-lagged questionnaire survey to collect 329 valid responses from international students at three universities in North, South, and East China. The research aims to investigate how GenAI use impacts students’ sociocultural adaptation, while examining the mediating roles of positive reappraisal and perceived empathy, as well as the moderating effect of AI anthropomorphism. The findings reveal that GenAI use is significantly positively associated with international students’ sociocultural adaptation. Positive reappraisal and users’ subjective perceived empathy mediate the relationship between GenAI use and sociocultural adaptation. Additionally, the degree of AI anthropomorphism positively moderates the relationships between GenAI use and both positive reappraisal and perceived empathy, enhancing the indirect effects of these mediating variables on the relationship between GenAI use and sociocultural adaptation. This study enriches the technological premises of cross-cultural adaptation for international students and provides GenAI-based intervention strategies for their educational management.

## 1 Introduction

In an era of increasing global mobility, international students often face significant sociocultural adaptation challenges when adjusting to new academic, social, and cultural environments (Wilczewski and Alon, 2023). China currently has the largest number of outbound students globally, with over 8 million Chinese students studying abroad in more than 100 countries and regions by 2023, according to data from China’s Ministry of Education (Li et al., 2024). In cross-cultural contexts, these students encounter complex sociocultural challenges, including academic pressure, daily life coordination, and interpersonal communication barriers (Cao and Meng, 2022). Sociocultural adaptation not only affects their academic performance but also influences the long-term development of their cross-cultural competencies and mental health. While existing research has explored factors such as social support, cultural distance, and individual differences (Lee and Ciftci, 2014; Zhu et al., 2022), most existing studies treat generative artificial intelligence (GenAI) merely as a tool for language learning or academic tasks (Wu and Yu, 2024; Chan and Hu, 2023), overlooking its potential as a cognitive-affective resource that interacts with users’ psychological processes. Additionally, these studies often analyze cognitive strategies and emotional experiences in isolation, neglecting their synergistic role in technology-mediated adaptation and failing to leverage the cognitive-affective personality system theory (CAPS) to uncover how GenAI activates this dual cognitive-emotional system (Kell, 2018). The rapid advancement of GenAI offers a new lens for understanding this process. As a pivotal technological tool in the digital age, GenAI is reshaping the cross-cultural adaptation pathways of international students through its capabilities in personalized learning support, task automation, and emotional interaction. However, current studies have yet to fully explore the mechanisms underlying this technology-driven adaptation. Sociocultural adaptation is fundamentally a dynamic process where individuals integrate cognitive and emotional resources to cope with their environment and regulate their psychology in foreign cultural contexts (Hobfoll, 2002). The CAPS and conservation of resources theory (COR) suggest that individuals tend to buffer stress by acquiring external resources. GenAI can be viewed as a digital adaptive resource that supports international students’ sociocultural adaptation in two key ways. Cognitively, GenAI may reduce information asymmetry and enhance task efficiency through functions like real-time information retrieval, academic writing assistance, and cultural rule interpretation (Wu and Yu, 2024), thereby mitigating negative perceptions of host-country socioculture. Emotionally, GenAI may provide emotional companionship and stress relief through emotion-perceptive algorithms, alleviating cross-cultural loneliness and anxiety (Pentina et al., 2023). Additionally, AI anthropomorphism, a feature of technological interfaces, may moderate the strength of GenAI’s effects, leading to differentiated adaptation outcomes. However, existing literature primarily focuses on GenAI’s instrumental applications in language learning and academic support, lacking systematic explanations and empirical tests of how it influences sociocultural adaptation through cognitive processing and emotional experiences. To bridge these gaps, drawing on CAPS and COR, this study seeks to answer the following research questions: How is GenAI use positively associated with international students’ sociocultural adaptation? What roles do positive reappraisal and perceived empathy play in this relationship? How does AI anthropomorphism moderate the impacts of GenAI use on positive reappraisal and perceived empathy? The findings not only expand the technological dimension of cross-cultural adaptation theory but also provide GenAI-based intervention strategies for international student education and management, carrying significant theoretical and practical implications.

## 2 Theory and hypotheses development

### 2.1 GenAI use and sociocultural adaptation

Sociocultural adaptation refers to international students’ ability to effectively navigate daily life, academic challenges, and interpersonal interactions in a new cultural environment. For these students, sociocultural adaptation not only influences academic performance but also shapes the long-term development of their cross-cultural competencies (Ward and Kennedy, 1999; Wilczewski and Alon, 2023). As an emerging technology, GenAI has the potential to reshape students’ adaptation processes by providing personalized learning experiences, automating administrative tasks, and supporting collaborative communication. Studies have shown that students perceive GenAI as a tool to enhance learning, foster creativity, and increase engagement (Qi et al., 2025). According to the COR, individuals tend to buffer acculturative stress and enhance adaptation capabilities by acquiring external resources (Hobfoll, 2002). GenAI-powered tools, such as chatbots and virtual tutors, offer interactive learning environments, instant feedback, and personalized learning pathways (Chen, 2024), helping students tackle academic challenges. Additionally, GenAI may provide basic life support related to daily needs—such as querying local transportation routes, recommending culturally appropriate dining venues, or interpreting accommodation contract clauses—through real-time data integration and natural language interaction (Dwivedi et al., 2024). These functions reduce adaptation stress caused by information asymmetry and enhance students’ sociocultural adaptation capabilities. Based on the above, we propose the following hypothesis:

*H1:* GenAI use is positively associated with international students’ sociocultural adaptation.

### 2.2 GenAI, positive reappraisal, perceived empathy, and sociocultural adaptation

Based on CAPS, information processing in individuals can occur through low-level, automatic affective systems or high-level, elaborative cognitive systems (Kell, 2018; Shiv and Fedorikhin, 1999). Drawing on COR, thus GenAI can be further conceptualized as a “digital resource buffer” that alleviates acculturative stress via instrumental and emotional support. COR explains how GenAI acts as a resource buffer, while CAPS reveals the cognitive-affective mechanisms through which these resources are internalized. This aligns with prior research showing that resource availability alone does not guarantee adaptive outcomes, the psychological processing of these resources is equally critical (Yoon and Lee, 2021; Bizri et al., 2025). In cross-cultural contexts, the use of GenAI (such as culturally adaptive chatbots or situational simulation assistants) may activate the dual processing of international students’ cognitive-affective systems through technological support.

Positive reappraisal refers to a cognitive strategy by which individuals reinterpret stressful situations to assign them positive meanings. Research has shown that environmental resources and levels of social support influence individuals’ positive reappraisal (Riepenhausen et al., 2022; Metts and Craske, 2023). As a critical cognitive aid in the digital age, AI use may also impact positive reappraisal. According to CAPS, adaptive behaviors rely on the cognitive processing and meaning-making of external information. GenAI provides personalized information support—such as cultural rule interpretation and academic task guidance—that reduces students’ uncertainty about unknown situations (Chan and Hu, 2023), making it easier for them to redefine cultural conflicts as “solvable learning tasks” rather than “threatening challenges.” Additionally, GenAI helps students develop cognitive habits of multiple perspectives by offering alternative explanatory frameworks (Borge et al., 2024), thereby mitigating the negative impacts of cultural conflicts and enhancing positive perceptions of stress. Furthermore, positive reappraisal yields positive behavioral outcomes for international students: it significantly reduces the negative emotional load of stressful situations (Brockbank and Feldon, 2024), prompting individuals to allocate psychological resources toward adaptive actions. Based on the above, we propose the hypothesis:

*H2a:* Positive reappraisal mediates the relationship between GenAI use and international students’ sociocultural adaptation.

Perceived empathy refers to users’ subjective emotional evaluation of whether AI demonstrates empathic capabilities, specifically their perception that AI can understand and respond to their emotional needs (Liu-Thompkins et al., 2022; Derakhshan et al., 2025). This construct is distinct from “true empathy” (which implies actual emotional intelligence in AI) and should not be conflated with satisfaction or utility value. Specifically, for international students, this involves their emotional evaluation of AI’s empathy ability, embodied in their perception of AI’s capacity to understand and respond to their emotional needs. As a generative technological tool, AI has the ability to engage with human thoughts, feelings, behaviors, and experiences (Liu-Thompkins et al., 2022). When GenAI interacts frequently with international students in daily life, students may view it as a collaborative partner, fostering emotional elements such as care and companionship (Derakhshan et al., 2025). Studies have found that adaptation anxiety and loneliness significantly negatively impact sociocultural adaptation (Kamalova et al., 2020; Brisset et al., 2010). GenAI may trigger automatic emotional responses through anthropomorphic interactions—such as using culturally appropriate greetings or detecting emotional signals in speech tone—to create emotional experiences of “being understood” and “being accepted” (Tran, 2020). This enhances students’ perceived empathy, thereby reducing anxiety and alienation in cross-cultural interactions (Zhang and Noels, 2024). For students who use AI extensively, they rely on AI to handle academic, daily life, and emotional issues. AI can recognize emotional vocabulary and tones in students’ input and generate empathic responses, leading to a perception of being understood. This reduces the emotional drain on students’ adaptive resources and enhances their sociocultural adaptation capabilities. Thus, we propose the hypothesis:

*H2b:* Perceived empathy mediates the relationship between GenAI use and international students’ sociocultural adaptation.

### 2.3 The moderating role of AI anthropomorphism

AI anthropomorphism refers to the act of attributing human characteristics to non-human entities, reflecting how closely AI’s interaction interface, language patterns, and emotional feedback mechanisms mimic human features (Pelau et al., 2021). This is a design-driven illusion of humanity rather than an intrinsic AI trait, influencing users’ cognitive-affective processes by creating perceived social similarity. CAPS highlights anthropomorphism’s role as a situational cue shaping cognitive-affective interaction, while COR frames it as an emotional resource buffering adaptation stress, thereby integrating dual mechanisms of cognitive processing and emotional support. From a CAPS perspective, anthropomorphism acts as a situational cue that enhances the credibility of GenAI’s cognitive guidance and emotional support, activating the integration of users’ cognitive and emotional processes (Kell, 2018). Simultaneously, COR theory frames anthropomorphism as an emotional resource that mitigates adaptation stress by providing companionship and trust, thereby preserving psychological energy (Hobfoll, 2002). AI with high anthropomorphic features may construct trustworthy scenarios of human-like interaction, making it easier for international students to internalize the cognitive frameworks it provides as their own positive reappraisal strategies. Specifically, individuals tend to trust agents with social similarity (Li and Suh, 2022), and the partner role created by anthropomorphic AI enhances the persuasiveness of its suggestions. This promotes students’ reinterpretation of cultural conflicts and amplifies the effectiveness of AI tool use. Additionally, at high levels of anthropomorphism, guiding suggestions provided during AI interactions are more readily accepted, effectively reducing students’ resistance (Polyportis and Pahos, 2025) and creating favorable conditions for the implementation of positive reappraisal strategies. Therefore, we propose the following hypothesis:

*H3a:* AI anthropomorphism moderates the positive relation between GenAI use and positive reappraisal, such that the relation is stronger at higher (vs. lower) levels of AI anthropomorphism.

From the perspective of emotional interaction mechanisms, high AI anthropomorphism may amplify the positive effect of AI use on perceived empathy by strengthening human-like emotional interaction features. Specifically, when AI has high anthropomorphic characteristics, students experience a stronger sense of being emotionally understood. This human-like interaction pattern triggers automatic emotional responses and fosters a resonance of acceptance (Walther et al., 2008). In contrast, low-anthropomorphism AI only provides functional support, lacking deep emotional processing in interactions and thus failing to activate users’ emotional resonance systems. From the lens of the Conservation of Resources Theory, highly anthropomorphic AI can be seen as a carrier of emotional resources, effectively replenishing the psychological energy expenditure of international students during cross-cultural adaptation (Ma et al., 2024). Its human-like emotional feedback mechanisms meet individuals’ basic needs for social connection by providing emotional support. In contrast, the neutral feedback of low-anthropomorphism AI lacks emotional warmth, unable to sustain emotional engagement and potentially leading to decreased perceived empathy. Therefore, we propose the following hypothesis:

*H3b:* AI anthropomorphism moderates the positive relation between GenAI use and perceived empathy, such that the relation is stronger at higher (vs. lower) levels of AI anthropomorphism.

Given the mediating roles of positive reappraisal and perceived empathy, as well as the moderating role of AI anthropomorphism, this study further proposes hypotheses about moderated mediation effects. Specifically, the dual paths through which AI use influences international students’ sociocultural adaptation—via positive reappraisal and perceived empathy—are moderated by the degree of AI anthropomorphism. When AI anthropomorphism is higher, the mediating effects of positive reappraisal and perceived empathy are strengthened. Based on this, we propose the following hypotheses:

*H4a:* AI anthropomorphism affect moderates the positive indirect relation between GenAI use and international students’ sociocultural adaptation via positive reappraisal, such that the indirect relation is stronger for higher (vs. lower) AI anthropomorphism.

*H4b:* AI anthropomorphism affect moderates the positive indirect relation between GenAI use and international students’ sociocultural adaptation via perceived empathy, such that the indirect relation is stronger for higher (vs. lower) AI anthropomorphism.

**Figure 1:**
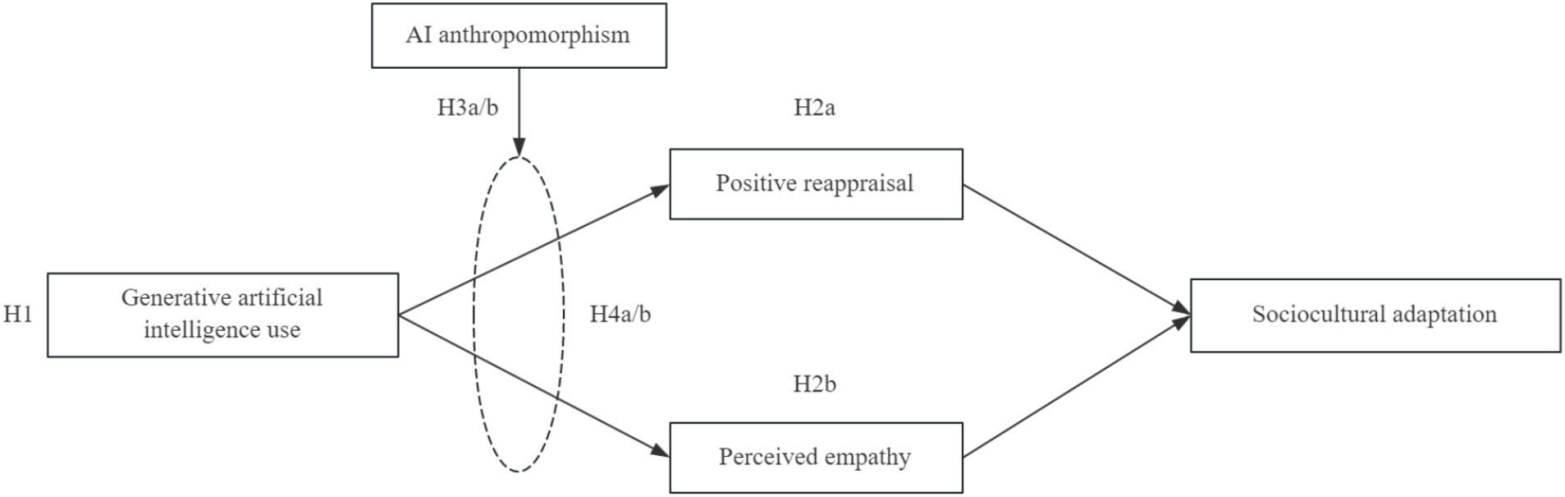
Research model.

The corresponding theoretical model is shown in Figure 1.

## 3 Materials and methods

### 3.1 Participants and procedures

This study recruited international students from three universities in North, South, and East China, as well as overseas students via online surveys. To protect participants’ privacy, all questionnaires were filled out anonymously. To mitigate common method variance, a three-stage longitudinal data collection method was employed, with surveys distributed at different time points over 2 months, spaced 1 week apart. Before distribution, participants were informed of the study’s purpose, and surveys were only administered to volunteers. Class teachers contacted participants to complete the questionnaires via the Wenjuanxing platform, and students who carefully filled out the surveys received a 10 RMB reward. Data were matched using participants’ registered ID numbers across stages. Specifically, Time 1 collected control variables (gender, age, education level, major, study duration, number of local friends), generative AI use, and AI anthropomorphism variables. A total of 400 questionnaires were distributed, yielding 373 valid responses (response rate: 93.3%). Time 2 surveyed positive reappraisal and perceived empathy among the same participants, resulting in 351 valid responses (response rate: 94.1%). Time 3 measured sociocultural adaptation, with 329 valid responses collected (response rate: 93.7%). Among the 329 questionnaires, 186 people were aged 18–22 (56.5%), 94 people were aged 23–26 (28.6%), 45 people were aged 27–30 (13.7%), and 4 people were over 31 years old (1.2%). There were 167 males, accounting for 50.8%, and 156 females, accounting for 49.2%. In terms of educational background, 203 people were undergraduates (61.7%), 122 people were postgraduates (37.1%), and 4 people were doctoral students (1.2%). In terms of majors, 68 people were in science and engineering (20.7%), 109 people were in humanities and social sciences (33.1%), and 152 people were in business (46.2%). In terms of study duration, 137 people studied for 1–6 months (41.6%), 98 people studied for 7–12 months (29.8%), 76 people studied for 13–24 months (23.1%), and 18 people studied for more than 24 months (5.5%). In terms of the number of local friends, 98 people had 0 local friends (29.8%), 132 people had 1–3 local friends (40.1%), 72 people had 4–6 local friends (24.9%), and 17 people had more than 7 local friends (5.2%).

### 3.2 Measures

GenAI use. The scale developed by Kanont et al. (2024) was used, consisting of 5 items, such as “Do you think you always use artificial intelligence to create text, images, or videos?” and “Do you think you will use artificial intelligence to support learning every time?” (*α* = 0.91).

Positive reappraisal. The Positive Reappraisal subscale from Zhu et al. (2007) revised Cognitive Emotion Regulation Questionnaire was adopted, including 4 items like “I think I can learn something from the situation” and “I look for the positive sides to the matter” (*α* = 0.93).

Perceived empathy. Yoon and Lee’s scale was used (Yoon and Lee, 2021), comprising 3 items such as “AI understands my specific needs” and “AI service has given me personalized attention” (*α* = 0.89).

Sociocultural Adaptation. Ward and Kennedy’s scale was employed (Ward and Kennedy, 1999), containing 12 items like “I have no difficulty using transportation” and “I can adapt to the local pace of life” (*α* = 0.85).

AI Anthropomorphism. The scale developed by used Kim and McGill (2011), including 3 items such as “AI looks like people” and “AI seems to have its own will” (*α* = 0.93).

Control variables. Age (1 = 18–22 years, 2 = 23–26 years, 3 = 27–30 years, 4 = 31 + years), gender (1 = male, 2 = female), education level (1 = undergraduate, 2 = masters, 3 = doctoral), academic discipline (1 = science/engineering, 2 = humanities/social sciences, 3 = business), study duration (1 = 1–6 months, 2 = 7–12 months, 3 = 13–24 months, 4 = 24 + months), and number of local friends (1 = 0, 2 = 1–3, 3 = 4–6, 4 = 7+) were included as control variables. These variables were selected based on prior research (Cao and Meng, 2022), which identified them as influencing international students’ behavioral attitudes, to ensure accurate examination of how generative AI use impacts their sociocultural adaptation.

### 3.3 Common method variance

Although this study employed a three-stage data collection method with a time lag, there is still a potential for common method bias due to all variable items being self-reported. Therefore, this study utilized the Unmeasured Latent Method Construct (ULMC) to assess common method bias (Richardson et al., 2009). The ULMC results showed that after placing all measured items onto a latent method factor, the six-factor structural model produced only minor changes compared to the five-factor structural model (ΔΧ^2^/df = 0.07, ΔCFI = 0.000, ΔTLI = 0.000, ΔRMSEA = 0.001, ΔSRMR = 0.016), and some fit indices even deteriorated, suggesting that adding a common method factor did not significantly improve the fit indices. Thus, there is no severe common method bias in this study.

### 3.4 Validity analysis

A structural equation model (SEM) was constructed using Mplus 8.0 to examine the discriminant validity of GenAI use, positive reappraisal, perceived empathy, sociocultural adaptation, and AI anthropomorphism. As shown in Table 1, the results indicated a five-factor model with good discriminant validity: *χ*^2^/df = 2.179, CFI = 0.976, TLI = 0.963, RMSEA = 0.058, and SRMR = 0.032.

**Table 1:** Results of the confirmatory factor analysis.

Model	Factors	*χ*^2^	*df*	*χ* ^2^ */df*	CFI	TLI	RMSEA	SRMR
Model a	GenAI; PR; PE; SA; AA	684.206	314	2.179	0.976	0.963	0.058	0.032
Model b	GenAI+ PR; PE; SA; AA	1167.838	318	3.641	0.897	0.883	0.072	0.063
Model c	GenAI+ PR + PE; SA; AA	1833.552	321	5.712	0.815	0.803	0.100	0.093
Model d	GenAI+ PR + PE + SA; AA	3101.769	323	9.603	0.752	0.740	0.133	0.112
Model e	GenAI+ PR + PE + SA + AA	3902.580	324	12.045	0.513	0.438	0.245	0.126

GenAI, Generative artificial intelligence use; PR, Positive reappraisal; PE, Perceived empathy; SA, Sociocultural adaptation; AA, AI anthropomorphism.

### 3.5 Correlation analysis

This study conducted descriptive statistical analysis and examined the correlation coefficients among variables, with results presented in Table 2. The findings showed that GenAI was positively correlated with international students’ sociocultural adaptation (r = 0.23, *p <* 0.01), positive reappraisal (r = 0.31, *p <* 0.01), and perceived empathy (r = 0.26, *p <* 0.01). Positive reappraisal was also positively correlated with sociocultural adaptation (r = 0.33, *p <* 0.01), as was perceived empathy with sociocultural adaptation (r = 0.21, *p <* 0.01). These results preliminarily supported the hypotheses.

**Table 2:** Mean, SD, correlations, and reliability.

Variables	1	2	3	4	5	6	7	8	9	10	11
1. Age	–										
2. Sex	0.03	–									
3. Edu	0.02	0.03	–								
4. Major	−0.01	−0.01	0.00	–							
5. Time abroad	−0.07	0.07	0.06	0.04	–						
6.local friends	0.13^*^	0.02	0.18^*^	0.02	0.15^*^	–					
7. GenAI	0.02	0.12^*^	0.19^*^	0.03	0.06	0.02	**0.72**				
8. PR	0.03	0.03	0.14^*^	−0.05	0.14^*^	0.16^*^	0.31^**^	**0.67**			
9. PE	−0.12^*^	−0.08	0.07	0.06	0.10	−0.08^*^	0.26^**^	0.03	**0.78**		
10. SA	−0.06	0.10	0.13^*^	−0.09	−0.12^*^	0.12^*^	0.23^**^	0.33^**^	0.21^**^	**0.61**	
11. AA	0.02	0.00	0.02	0.01	0.02	0.10	0.08	0.17^**^	0.15^*^	0.10	**0.81**
*M.*	1.73	3.17	1.73	1.12	3.35	1.71	3.14	3.55	1.79	2.23	3.97
*S. D.*	0.46	0.84	0.61	0.73	0.72	0.58	0.87	0.67	0.96	0.84	1.12

**Correlation is significant at the 0.01 level (2-tailed). *Correlation is significant at the 0.05 level (2-tailed). The bold values on the diagonal indicated the AVE. GenAI, Generative artificial intelligence use; PR, Positive reappraisal; PE, Perceived empathy; SA, Sociocultural adaptation; AA, AI anthropomorphism.

## 4 Results

### 4.1 Direct and indirect effect

This study tested the hypotheses using Mplus 8.0, with results summarized in Tables 3, 4. The findings showed that GenAI was positively associated with international students’ sociocultural adaptation (*γ* = 0.27, *p <* 0.05). GenAI was also positively related to positive reappraisal (*γ* = 0.24, *p <* 0.05), and positive reappraisal was positively linked to sociocultural adaptation (*γ* = 0.43, *p <* 0.05). Bootstrap analysis revealed that GenAI had an indirect effect on sociocultural adaptation through positive reappraisal, with an effect size of 0.147 (S. E. = 0.04, 95% CI = [0.052, 0.171]), supporting Hypotheses H1 and H2a. Additionally, GenAI was positively correlated with perceived empathy (*γ* = 0.19, *p <* 0.05), and perceived empathy was positively correlated with sociocultural adaptation (*γ* = 0.34, *p <* 0.05). Bootstrap results indicated an indirect effect of GenAI on sociocultural adaptation via perceived empathy, with an effect size of 0.124 (S. E. = 0.03, 95% CI = [0.023, 0.137]), supporting Hypothesis H2b.

**Table 3:** Results of path coefficient.

Variables	PR			PE			SA		
	Estimate	S. E.	*P*	Estimate	S. E.	*P*	Estimate	S. E.	*P*
Age	−0.02^*^	0.01	0.036	−0.02^*^	0.01	0.037	−0.02	0.02	0.089
Sex	0.01	0.05	0.067	0.00	0.09	0.277	0.01	0.04	0.092
Edu	0.05	0.03	0.039	0.05^*^	0.03	0.013	0.02^*^	0.01	0.044
Major	0.15^*^	0.11	0.045	0.16^*^	0.08	0.024	0.04^*^	0.02	0.027
Time abroad	−0.11^*^	0.12	0.037	−0.07	0.07	0.071	0.03	0.03	0.064
Local friends									
Independent variable									
GenAI	0.24^*^	0.14	0.036	0.19^*^	0.17	0.024	0.27^*^	0.05	0.012
Mediator variables									
PR							0.43^*^	0.14	0.018
PE							0.34^*^	0.17	0.033
Moderator variable									
AA	0.23^*^	0.09	0.017	0.25^**^	0.04	0.002	0.08	0.06	0.085
Interaction									
GenAI× AA	0.38^***^	0.04	0.000	0.27^**^	0.08	0.001	0.24^**^	0.12	0.007
Residual variance	0.69^***^	0.04	0.000	0.42^***^	0.03	0.000	0.71^***^	0.06	0.000
*R^2^*	29%			21%			36%		

***P < 0.001, **p < 0.01, *p < 0.05. GenAI, Generative artificial intelligence use; PR, Positive reappraisal; PE, Perceived empathy; SA, Sociocultural adaptation; AA, AI anthropomorphism.

**Table 4:** Results of mediating effect analysis.

Mediating effect	Estimate	S. E.	95%CI
GenAI → Positive reappraisal → Sociocultural adaptation	0.147	0.04	[0.052, 0.171]
GenAI → Perceived empathy → Sociocultural adaptation	0.124	0.03	[0.023, 0.137]
Total effect	0.271	0.04	[0.194, 0.275]

### 4.2 Moderation analysis

**Figure 2:**
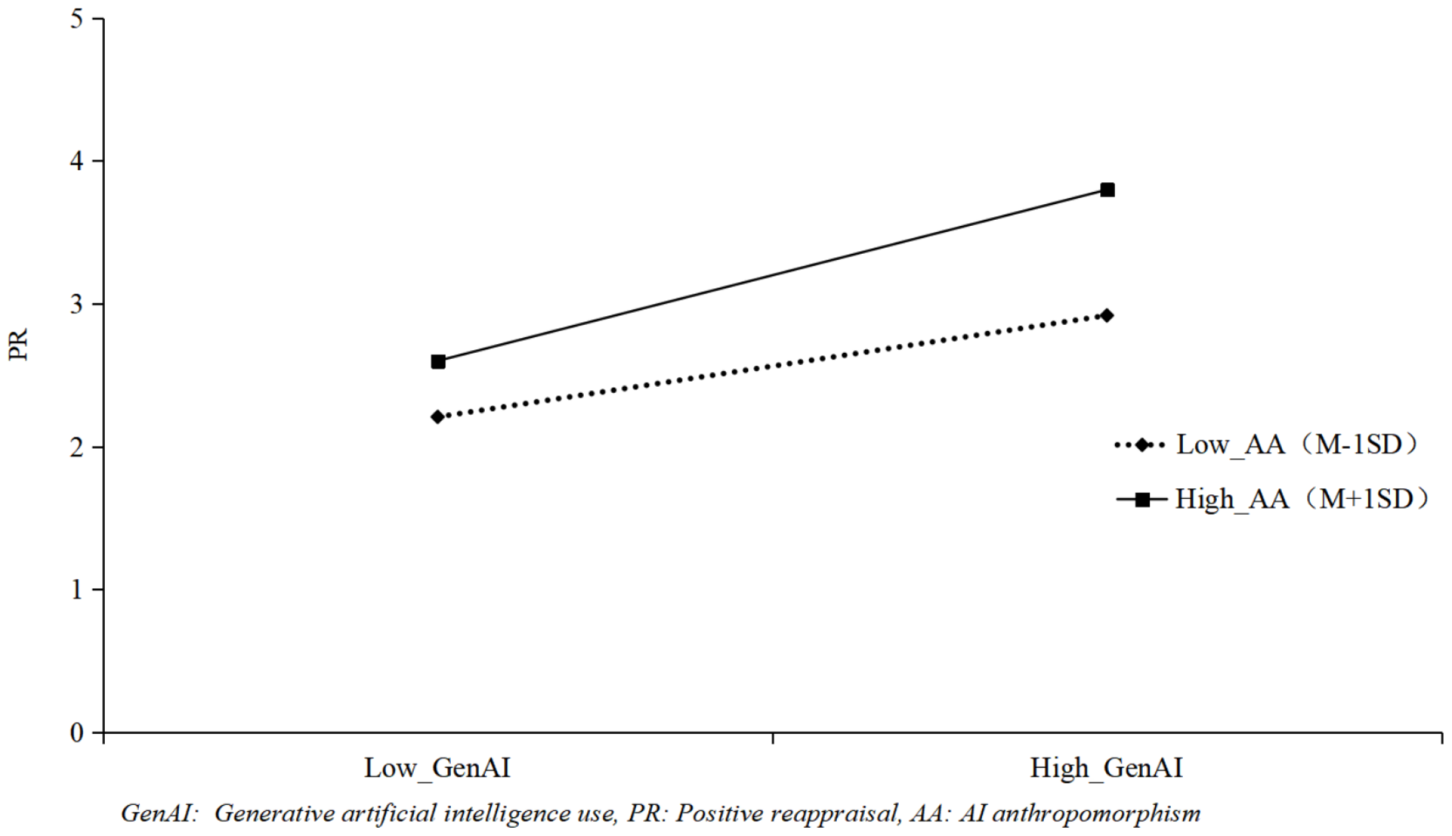
The moderating role of AA between GenAI and PR.

**Figure 3:**
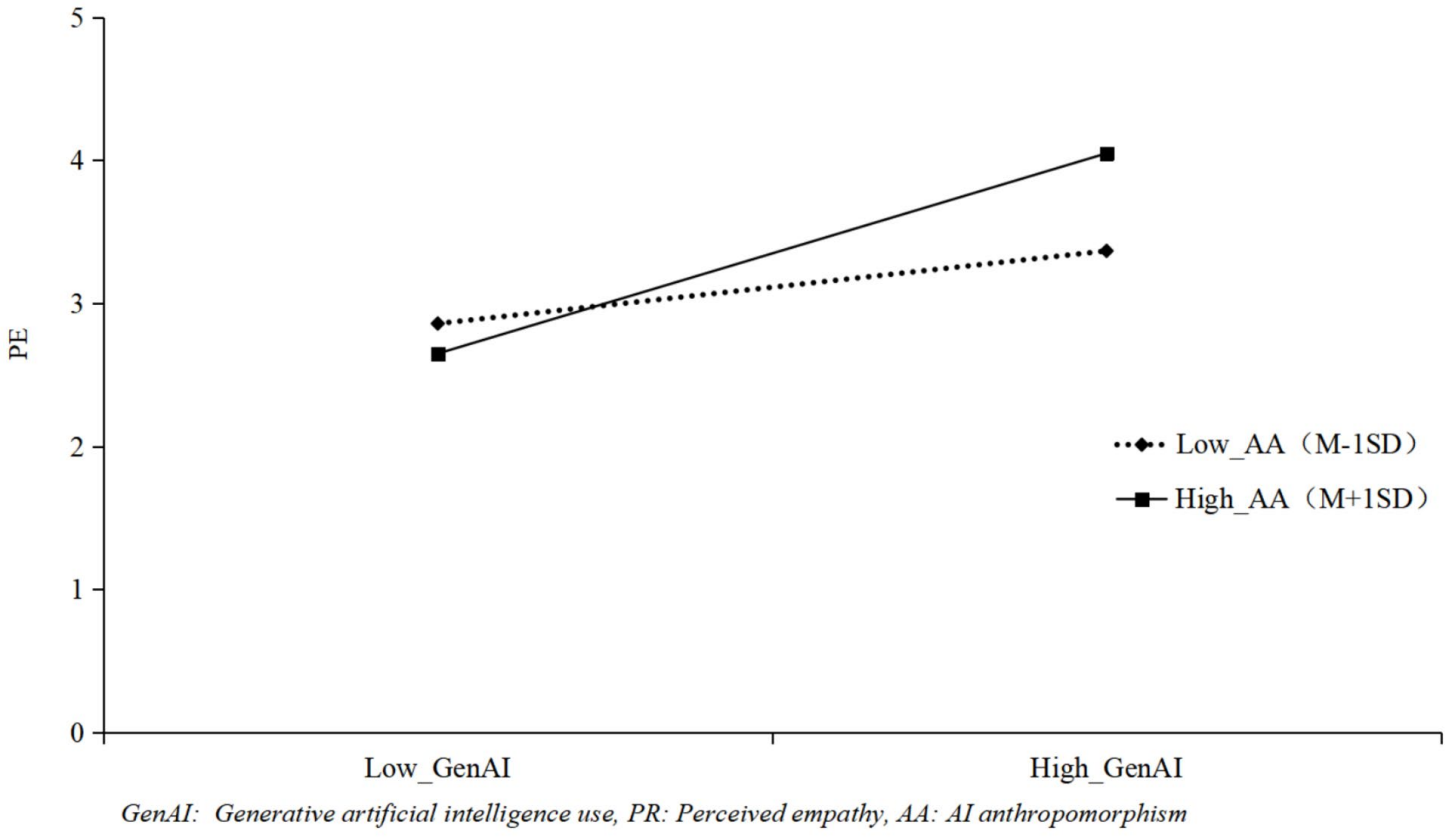
The moderating role of AA between GenAI and PE.

As shown in Table 3, the interaction term of GenAI and AI Anthropomorphism had a positive effect on positive reappraisal (*γ* = 0.38, *p <* 0.001) and perceived empathy (*γ* = 0.25, *p <* 0.01). To visualize the moderating role of AI anthropomorphism, Figures 2, 3 were plotted. High AI anthropomorphism was defined as one standard deviation above the mean, and low AI anthropomorphism was defined as one standard deviation below the mean. Figure 2 indicated that the positive effect of GenAI on positive reappraisal was stronger when AI anthropomorphism was high, supporting Hypothesis H3a. Using the same approach, Figure 3 showed that the positive effect of GenAI on perceived empathy was more pronounced under high AI anthropomorphism, supporting Hypothesis H3b.

### 4.3 Testing the moderated mediation effects

To test the moderated mediation effects, this study performed 5,000 bootstrap samples using Mplus 8.0 to analyze the conditional indirect effects. As shown in Table 5, under high AI anthropomorphism, the indirect effect of GenAI on international students’ sociocultural adaptation through positive reappraisal was higher, with a point estimate of 0.27 (95% CI = [0.163, 0.340]), and the indirect effect was significant. Under low AI anthropomorphism, this indirect effect was lower, with a point estimate of 0.12 (95% CI = [0.054, 0.087]), also significant, supporting Hypothesis H4a. Similarly, for the indirect effect through perceived empathy, high AI anthropomorphism yielded a higher indirect effect (Estimate = 0.17, 95% CI = [0.033, 0.121]), while low AI anthropomorphism resulted in a lower indirect effect (Estimate = 0.08, 95% CI = [0.004, 0.096]). Both indirect effects were significant, providing support for Hypothesis H4b.

**Table 5:** Results of the moderated mediation.

Moderator variable AI anthropomorphism	GenAI → Positive reappraisal → Sociocultural adaptation			GenAI → Perceived empathy → Sociocultural adaptation		
	Estimate	S. E.	95% CI	Estimate	S. E.	95% CI
High (mean + 1SD)	0.27	0.04	[0.163, 0.340]	0.17	0.03	[0.033, 0.121]
Low (mean - 1SD)	0.12	0.06	[0.054, 0.087]	0.08	0.04	[0.004, 0.096]
High vs. low	0.15	0.03	[0.027, 0.106]	0.09	0.01	[0.002, 0.061]

## 5 Discussion

The theoretical contributions of this study primarily lie in uncovering the mechanisms through which GenAI technology influences international students’ sociocultural adaptation and expanding the CAPS and COR. First, differing from traditional cross-cultural adaptation research that relies on factors such as social support and cultural distance (Lee and Ciftci, 2014; Zhu et al., 2022), this study introduces GenAI as a technological antecedent to explore its impact on students’ sociocultural adaptation. By doing so, it enriches the role of technological tools in cross-cultural adaptation and responds to the call for integrating AI technologies into educational settings (Wang et al., 2023). Second, based on CAPS and COR, this study constructs the connotations and pathways of resources in cross-cultural adaptation. The findings reveal that GenAI influences students’ sociocultural adaptation through dual channels: a cognitive pathway (positive reappraisal) and an emotional pathway (perceived empathy). This not only validates the unique value of technological tools as digital adaptive resources (Adeshina, 2024) but also provides empirical support for applying CAPS and COR in technological contexts. It offers a new paradigm for integrating cognitive and emotional mechanisms in technology-driven adaptation, enriching research on how technological interventions shape psychological processes. Finally, by introducing AI anthropomorphism as a moderating variable, this study reveals the differential effects of technological interface characteristics on users’ psychological experiences. Existing literature has focused mostly on the functional attributes of GenAI, neglecting the reinforcing mechanisms of the social attributes of interaction design on adaptation outcomes (Bandi et al., 2023). This study finds that highly anthropomorphic AI enhances students’ positive reappraisal tendencies and perceived empathy through human-like interactions, significantly amplifying the facilitative effect of GenAI use on adaptation capabilities. This conclusion expands the theoretical boundaries of interactions between technological features and user psychology, indicating that the social-like attributes of technology can activate individuals’ dual systems of cognitive processing and emotional experience, thereby enriching the theoretical connotations of cross-cultural adaptation in the digital age.

The study also holds practical implications for educational management. First, to improve students’ proficiency in using GenAI, foundational courses on GenAI operations should be offered, integrating ethical modules on data privacy, algorithmic transparency, and cultural bias mitigation with modular instruction to help international students master core functions (Pesovski et al., 2024). Educators should guide students to view technology as a collaborative assistant while implementing stratified training for teachers, such as workshops on “AI-Enhanced Cross-Cultural Pedagogy,” to equip faculty with skills to design ethically aligned GenAI interventions (Biagini, 2025). Second, leveraging the dual cognitive-emotional pathways, educators can design AI-driven cultural conflict simulation labs where inputting real-world scenarios generates multi-solution frameworks to cultivate problem-solving cognitive patterns. To enhance emotional support, GenAI tools should incorporate dynamic adaptation monitoring, using sentiment analysis to detect signs of stress and trigger timely teacher interventions. Meanwhile, integrating a “cultural fit plugin” into GenAI interfaces will allow students to customize interaction styles, strengthening perceived empathy. Lastly, technology developers and institutions must collaborate to build interdisciplinary ethical design frameworks (Patel et al., 2019), co-creating anthropomorphic features that reflect cultural nuances, such as adjusting greeting protocols for religious holidays or adapting feedback tones to align with collectivist values (Kim et al., 2025). Additionally, establishing interuniversity GenAI resource hubs to share localized cultural datasets will ensure tools are contextually relevant. These strategies collectively form a human-AI synergistic ecosystem, where ethical guardrails, cognitive empowerment, and culturally attuned emotional support converge to enhance international students’ sociocultural adaptation.

## 6 Limitations and future research

Although this study has made valuable explorations, several limitations remain that warrant further improvement in future research. First, regarding the research design, while two data collection methods were employed, common method bias could not be fully avoided. Future studies could adopt a mixed approach combining self-assessment and peer assessment to collect data, reducing reliance on single-source responses. Second, the control variables used in this study were relatively basic, and potential external factors were not fully considered. To address this, future research might incorporate a broader range of control variables, such as cultural intelligence, AI trust, and the density of social support networks. Third, contextual experiments could be used to test the causal relationships between variables, providing stronger evidence for the proposed mechanisms. Finally, the sample in this study primarily focused on Chinese international students. Although this study reported scale reliability, the adopted scales were all directly translated from established English-language instruments without cultural adaptation or pilot testing for the specific population of international students at Chinese universities. Measurement bias may arise due to cultural background differences. Expanding the sample to include diverse cultural backgrounds, such as Western students in non-English-speaking countries or non-Chinese international students, would help validate the cross-cultural generalizability of the theoretical model. Additionally, this study did not account for potential cultural biases in AI interface design, such as how anthropomorphic features might be perceived differently across collectivist and individualistic cultures. For instance, East Asian students may prioritize functional utility over human-like interaction (Li and Suh, 2022), while Western users might associate anthropomorphism with emotional support (Pelau et al., 2021). Future research could employ experimental designs to manipulate AI anthropomorphism levels across diverse cultural samples, using longitudinal interventions to track how sustained, culturally adapted GenAI use influences sociocultural adaptation trajectories.

## Data Availability

The original contributions presented in the study are included in the article/supplementary material, further inquiries can be directed to the corresponding author.
